# Loss of angiotensin II receptor expression in dopamine neurons in Parkinson’s disease correlates with pathological progression and is accompanied by increases in Nox4- and 8-OH guanosine-related nucleic acid oxidation and caspase-3 activation

**DOI:** 10.1186/s40478-015-0189-z

**Published:** 2015-02-03

**Authors:** W Michael Zawada, Robert E Mrak, JoAnn Biedermann, Quinton D Palmer, Stephen M Gentleman, Orwa Aboud, W Sue T Griffin

**Affiliations:** Department of Geriatrics, The Reynolds Institute on Aging, University of Arkansas for Medical Sciences, 629 Jack Stephens Drive, Little Rock, AR 72205 USA; Department of Neurology, Alzheimer’s Research and Clinical Program, University of Colorado Denver, Aurora, CO 80045 USA; Department of Pathology, University of Toledo, Toledo, OH 43614 USA; Department of Medicine, Imperial College London, London, W6 8RP UK; Geriatric Research Education Clinical Center, Central Arkansas Veterans Health System, Little Rock, AR 72205 USA

**Keywords:** Angiotensin, Dopamine neuron, NADPH oxidase, Nox, Nigrosome, Oxidative stress, Parkinson’s, RAS, Substantia nigra

## Abstract

**Background:**

In rodent models of Parkinson’s disease (PD), dopamine neuron loss is accompanied by increased expression of angiotensin II (AngII), its type 1 receptor (AT1), and NADPH oxidase (Nox) in the nigral dopamine neurons and microglia. AT1 blockers (ARBs) stymie such oxidative damage and neuron loss. Whether changes in the AngII/AT1/Nox4 axis contribute to Parkinson neuropathogenesis is unknown. Here, we studied the distribution of AT1 and Nox4 in dopamine neurons in two nigral subregions: the less affected calbindin-rich matrix and the first-affected calbindin-poor nigrosome 1 of three patients, who were clinically asymptomatic, but had nigral dopamine cell loss and Braak stages consistent with a neuropathological diagnosis of PD (prePD). For comparison, five clinically- and neuropathologically-confirmed PD patients and seven age-matched control patients (AMC) were examined.

**Results:**

AT1 and Nox4 immunoreactivity was noted in dopamine neurons in both the matrix and the nigrosome 1. The total cellular levels of AT1 in surviving dopamine neurons in the matrix and nigrosome 1 declined from AMC>prePD>PD, suggesting that an AngII/AT1/Nox4 axis orders neurodegenerative progression. In this vein, the loss of dopamine neurons was paralleled by a decline in total AT1 per surviving dopamine neuron. Similarly, AT1 in the nuclei of surviving neurons in the nigral matrix declined with disease progression, i.e., AMC>prePD>PD. In contrast, in nigrosome 1, the expression of nuclear AT1 was unaffected and similar in all groups. The ratio of nuclear AT1 to total AT1 (nuclear + cytoplasmic + membrane) in dopamine neurons increased stepwise from AMC to prePD to PD. The proportional increase in nuclear AT1 in dopamine neurons in nigrosome 1 of prePD and PD patients was accompanied by elevated nuclear expression of Nox4, oxidative damage to DNA, and caspase-3-mediated cell loss.

**Conclusions:**

Our observations are consistent with the idea that AngII/AT1/Nox4 axis-mediated oxidative stress gives rise to the dopamine neuron dysfunction and loss characteristic of the neuropathological and clinical manifestations of PD and suggest that the chance for a neuron to survive increases in association with lower total as well as nuclear AT1 expression. Our results support the need for further evaluation of ARBs as disease-modifying agents in PD.

## Introduction

The renin-angiotensin system (RAS) in the brain is physically separated from the peripheral RAS by the blood–brain barrier (BBB) [1]. Because it regulates blood pressure and volume, RAS in the periphery is especially important for survival in the case of hemorrhage [2]. For this, circulating AngII binds to its type 1 receptor (AT1) on vascular smooth muscle cells [3] to induce vasoconstriction. The presence of a brain specific RAS and its potential as a regulator of the brain’s response to stress has been demonstrated in rodent [4], primate, and human brain [5]. Coordinated synthesis of AngII and AT1 in both neurons and glia in adult rat brain coincides with localization of AT1 on endolysosomes [6], on mitochondria, and in nuclei [7]. The first hint of a nuclear-delimited function of AngII came in the early 1970s, when trafficking of radiolabelled AngII into the nuclear compartment of smooth muscle cells was first reported, suggesting that nuclear AngII/AT1 signaling influences transcription [8]. In addition, AngII binding to AT1 in the mature brain contributes to: *i*) the maintenance of blood pressure [9]; *ii*) calcium-dependent hypothalamic neuron depolarization [6]; and *iii*) dopamine release from dopaminergic neurons [10,11]. On the other hand, AngII binding to AT1 during brain development favors: *iv*) neurogenesis [12] and *v*) differentiation of dopamine neurons [13].

The original link between AngII/AT1 and dopaminergic neurotransmission was established in the late 1970s by Simonnet and Giorguieff-Chesselet in a report on AngII-evoked release of dopamine from rat striatal slices [10]. Subsequently, autoradiography revealed AT1 binding sites in human nigral dopaminergic cell bodies and in the striatum in dopaminergic terminals [14,15]. These findings raised, for the first time, the possibility of the existence of a functionally active, self-contained AngII/AT1 system in dopamine neurons in substantia nigra that might be relevant to Parkinson’s disease. Indeed, studies in rodent models of Parkinson’s demonstrated that angiotensin receptor blockers (ARBs) such as losartan protect dopamine neurons from the degenerative effects of 1-methyl-4-phenyl-1,2,3,6-tetrahydropyridine (MPTP) in mice [4,16] and 6-hydroxydopamine (6-OHDA) in rats [17,18]. The neurodegenerative consequences of the AT1 activation might come from AT1-mediated stimulation of a multimeric NADPH oxidase (Nox) for production of excessive levels of superoxide, as we demonstrated for Nox2 in cultured rat dopaminergic cells [19] and for Nox4 in murine neural stem cells [12].

As rat dopamine neurons demonstrate an increase in the numbers of AT1 in response to 6-OHDA treatment [17] as well as with increased age, we posit here that elevation of expression of AT1 presages dopamine neuron dysfunction and loss. In parallel to these increases in the AT1 following intrastriatal 6-OHDA injection, p47 and Nox2, which are cytoplasmic and catalytic Nox subunits, respectively, are also elevated; thus, Nox is activated and oxidative damage to macromolecules ensues [20]. These responses are not limited to 6-OHDA models, as we have reported that a selective dopaminergic neurotoxin, 1-methyl-4-phenylpyridinium (MPP+), elevates expression of Nox2 and promotes Nox2-dependent superoxide generation in a midbrain-derived rat dopaminergic cell line and that these pathogenic effects are reduced by losartan [19]. These findings are consistent with a role for AngII/AT1/Nox-mediated oxidative stress in the initiation and progression of neurodegeneration in neurotoxin-induced Parkinson models. Our earlier report on the expression of p47 and Nox2 in dopaminergic neurons in the adult human substantia nigra [19], together with a demonstration in a human brain by Garrido-Gill and colleagues of nigral intraneuronal RAS [5], support the notion of a direct link between AngII/AT1 and Nox/superoxide generation in human dopamine neurons. Until now, there existed only a single report suggesting that in humans the response of the brain RAS to degenerative conditions results in a change in AT1 numbers. Although the phenotypic identity of the neural cells examined was not identified by this 1996 report by Ge and Barnes [21], they did report that relative to that present in age-, sex-, and post-mortem interval-matched neurologically normal individuals, there is a marked reduction in AngII binding to AT1 in the caudate, putamen, and substantia nigra in brains of Parkinson patients.

In order to identify and characterize AT1-related changes as a function of neurological and neuropathological changes characteristic of PD, we studied the distribution and levels of expression of AT1 in substantia nigra of three groups of patients: those who had neuropathological changes consistent with PD, but were asymptomatic as to motor dysfunction (pre-PD); those with frank PD; and patients of similar ages who died neurologically and neuropathologically intact (AMC). For a comparison of data from these cases, we developed a semi-quantitative immunofluorescence microscopy-based strategy for systematic analysis of the AT1 within subdivisions of the substantia nigra with regard to its steady state levels as well as its specific subcellular location in dopamine neurons in nigral tissue sections. The rationale for examining the matrix and nigrosome 1 subdivisions arose from earlier demonstrations by Damier and colleagues that human substantia nigra is compartmentalized into distinct zones termed nigrosomes 1–5 (calbindin poor) and matrix (calbindin rich) [22] and that the nigrosome 1 is the site of the earliest loss of dopamine neurons in Parkinson’s [23]. Reasoning that these nigral compartments might engender differential changes to the AT1-related pathogenic pathway, we examined these distinct nigral regions and found that the whole-cell levels of AT1 in dopamine neurons were gradually reduced in both nigral matrix and nigrosome 1 in a pattern indicative of a disease-related progression (AMC>prePD>PD). In contrast to the attendant and similar losses of AT1 in dopamine neurons and their nuclei in neurons of the nigral matrix, the nuclear AT1 profile in neurons of nigrosome 1 differed distinctly, i.e., the ratio of nuclear/total neuron AT1 expression was elevated with disease progression. Disease progression was also associated with increased levels of Nox4, StressMarq-detected oxidative damage to nucleic acids, and active caspase-3 in the nuclei of dopamine neurons.

## Materials and methods

### Case selection and neuropathological assessment

All brain tissues (n = 15) examined were from the Human Brain Bank at the Reynolds Institute on Aging at the University of Arkansas for Medical Sciences. Specific cases were selected using two criteria: *i)* presence or absence of motor symptoms characteristic of PD and *ii)* presence or absence of depigmentation of the nigra – an index of dopamine neuron survival. Patients who were presymptomatic (i.e., showed no motor symptoms), but displayed a detectable depigmentation of the nigra as well as α-synucleinopathy identified using BrainNet Europe Consortium staging protocols [24,25], i.e., Braak stage 6 (ages 69–79, 3 males) were classified as prePD patients. Patients with advanced disease (evident motor symptoms) combined with severe depigmentation of the nigra and Braak stage 6 (ages 66–85, 3 males, 2 females) were classified as PD. Neurologically and neuropathologically normal patients of similar age (Braak stage 0, ages 67–87, 6 males, 1 female) were used as age-matched controls (AMC) (Table 1). Autopsy tissues studied are exempt from Institutional Review Board (IRB) review under 46.101 5(b), and this study was approved as an exempt study by the University of Arkansas IRB.

**Table 1:** **Age, gender, clinical diagnosis, cause of death, post-mortem interval, and brain weight for control, prePD, and PD cases**

**All prePD and PD cases are α-synuclein Braak stage 6**						
**Case**	**Group**	**Age (y)/ gender**	**Clinical diagnosis**	**Cause of death**	**PMI (h)**	**Brain weight (g)**
1	AMC	67/M	Leukemia	Pneumonia	5.5	1450
2	AMC	78/M	Atherosclerosis	Pulmonary Embolism	19.0	1070
3	AMC	87/F	Atherosclerosis/diabetes	Renal failure	13.0	NA
4	AMC	85/M	Adenocarcinoma	Metastasis	11.0	1210
5	AMC	76/M	Atherosclerosis/diabetes	Heart failure	3.0	1480
6	AMC	87/M	Atherosclerosis	Aneurysm rupture	5.0	1375
7	AMC	79/M	Atherosclerosis	Heart failure	22.5	1550
8	PrePD	79/M	AD	Pneumonia	14.5	1100
9	PrePD	86/M	COPD	pneumonia	20.5	1175
10	PrePD	69/M	Primary aphasia	Unknown	<12.0	1475
11	PD	85/M	PD/dementia	Heart failure	5.5	NA
12	PD	76/M	PD/dementia	Pneumonia	7.0	1490
13	PD	84/M	PD/dementia	Unknown	1.5	1250
14	PD	66/F	PD/dementia	Unknown	10.0	1050
15	PD	77/F	PD/AD	Pneumonia	5.5	1075

Abbreviations: *COPD* chronic obstructive pulmonary disease and *PMI* post-mortem interval.

### Tissue preparation, immunohistochemical detection of Calbindin D28k, and immunohistofluorescence

**Figure 1:**
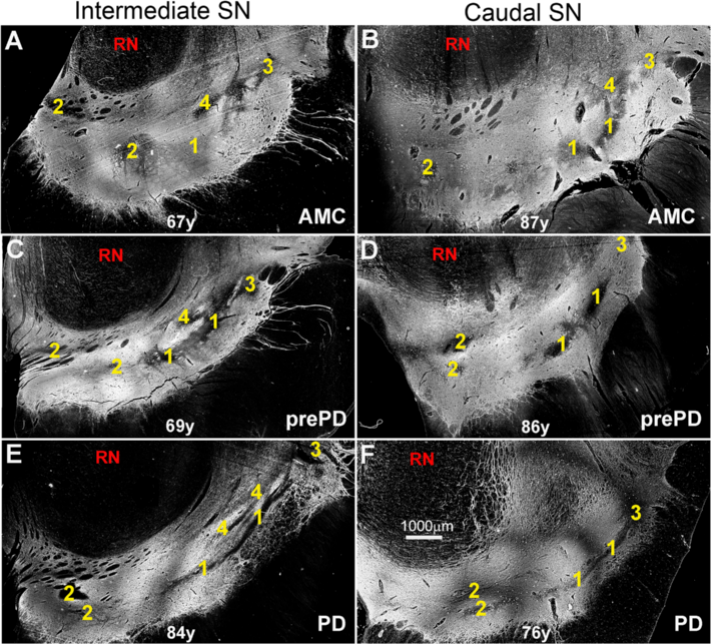
**Nigrosomes at specific rostrocaudal levels of the adult human SN.** Nigrosomes were identified in all 15 patients examined by Calbindin D28k immunohistochemistry in the intermediate **(A**, **C**, and **E)** and caudal (**B**, **D**, and **F**) SN. In these negative images, nigrosomes are depicted as dark areas (*i.e.,* calbindin-poor) and numbered 1–4. Matrix is calbindin-rich and appears white. We have examined one AOI for nigrosome 1 and another AOI for matrix for each case studied. Substantia nigra (SN); Red nucleus (RN). Scale bar is 1000 μm.

Brains were fixed in formalin (no methanol) for two weeks prior to further processing. Formalin-fixed midbrain was blocked in a transverse plane and paraffin-embedded prior to cutting 7 μm-thick sections of the substantia nigra at its intermediate to caudal levels and processed as previously described [26]. The intermediate substantia nigra was defined as that coinciding with the exit of Cranial Nerve III. All antibodies used, clone (m = monoclonal) or type (p = polyclonal), recognized epitope(s), methods for unmasking epitopes, dilutions, and procurement sources are listed in Table 2. The primary antibody for AT1 detection was a goat IgG from Santa Cruz (Cat# sc-1173-G). For each case, at least six sections from substantia nigra were cut, and at least one section immunostained for each of the following: *i*) TH/AT1/DAPI; *ii*) Nox4/AT1/DAPI; *iii*) StressMarq/AT1/DAPI; *iv*) caspase-3/TH/DAPI; *v*) α-synuclein; and *vi*) calbindin D28k. For select AMC, prePD, and PD cases additional sections were cut and processed for co-immunodetection of AT1 with one of the following GFAP, Iba1, Nup62, Pan-Neuronal whole neuron marker, or S100B. Calbindin D28k immunohistochemistry was used to identify nigrosomes in all 15 patients examined. Photomicrographs demonstrating position of nigrosomes 1–4, located in intermediate and caudal substantia nigra are identifiable as dark areas, *i.e.,* calbindin-poor regions adjacent to bright areas that are calbindin rich (Figure 1).

**Table 2:** **Antibodies used and antigen unmasking procedures employed **

**Antibody**	**Clone/type**	**Epitope**	**Unmasking**	**Dilution**	**Source**
α-synuclein	42, Mouse IgG1	Rat α-synuclein 15-123	70% Formic acid for 10 min	1:500	Becton-Dickenson
AT1	p Goat	Synthetic peptide mapping @ N-terminus of human AT1	Microwave in 10 mM citrate buffer pH 6 for 30 min	1:100	Santa Cruz
Calbindin D28k	p Rabbit	Rat calbindin D28k	Microwave in 10 mM citrate buffer pH 6 for 30 min	1:500	Swant
DNA/RNA damage	m Mouse 15A3	8-hydroxy-guanosine-BSA and -casein conjugates	Microwave in 10 mM citrate buffer pH 6 for 30 min	1:100	StressMarq
GFAP	p Chicken	Bovine full length native protein	Trypsin @37°C for 10 min	1:1000	Aves Labs
Iba1	p Rabbit	Synthetic peptide of C-terminus of Iba1 conserved between humans, rats, and mice	Microwave in 10 mM citrate buffer pH 6 for 30 min	1:400	Wako
Nup62	m Mouse	Human nucleoporin 62 aa. 24-178	Microwave in 10 mM citrate buffer pH 6 for 30 min	1:50	BD Transduction Laboratories
Pan-Neuronal	m Mouse	Whole neuron marker	Microwave in 10 mM citrate buffer pH 6 for 30 min	1:500	Millipore
Nox4	m Rabbit	Synthetic peptide within NADPH binding domain of human Nox4 (Q9NPH5)	Microwave in 10 mM citrate buffer pH 6 for 30 min	1:200	Abcam
S100B	Rabbit	35HT3-10	Trypsin @37°C for 10 min	1:1000	Dr. Linda Van Eldik
Tyrosine hydroxylase	p Rabbit	Synthetic peptide mapping @ N-terminus of human TH	Microwave in 10 mM citrate buffer pH 6 for 30 min	1:1000	Pel-Freez

Abbreviations: *m* monoclonal and *p* polyclonal.

Sections that were used for immunohistofluorescence were treated with 0.1% Sudan Black B (Sigma) in 70% ethanol to block lipofuscin autofluorescence. All immunoreactions were by overnight incubation at room temperature. Appropriate Alexa Fluor-tagged secondary antibodies were diluted in antibody diluent 1:200 (DAKO), and sections were incubated for 60 minutes, washed three times for 5 minutes each in distilled water, and coverslipped with prolong Gold with DAPI (Invitrogen) for DNA/nuclear staining. For each set of slides, one no primary antibody control section was treated exactly the same, including the secondary antibody. On two occasions, prior to immunohistofluorescence procedure on human SN sections, additional controls were run by pre-absorbing primary antibody against AT1 at 4°C overnight with AT1 blocking peptide (Santa Cruz Cat# sc-1173 P) against which the primary antibody was raised. The weight ratio of the primary AT1 antibody to the AT1 blocking peptide that totally prevented detection of the AT1 in SN was 1:50 (0.5 μg antibody:25 μg blocking peptide). At ratios of 1:20 and 1:10, the blocking peptide was also effective in lowering the intensity of AT1 fluorescence, but to a lesser extent than the complete AT1 signal blockade seen at the antibody to peptide ratio of 1:50 (data not shown). To confirm the staining pattern obtained with the primary anti-AT1 antibody from Santa Cruz, we have performed immunohistofluorescent staining of select sections from human brain with an alternative primary anti-AT1 antibody made in goat (Sigma Cat# SAB2500038).

### Semiquantitative assessment of AT1 in nigral TH-immunoreactive dopamine neurons

The expression of AT1 in TH-immunoreactive dopamine neurons of human substantia nigra was quantified using similar methods to those that we have previously described in different cell types including S100B- and GFAP-immunoreactive astrocytes and Iba1-immunoreactive microglia [26-28]. In the current study, each of the nigral sections examined contained both the calbindin poor nigrosome 1 region as well as the calbindin rich matrix (Figure 1). In each nigral section, the nigrosome 1 and matrix regions were identified based on the level of calbindin D28k immunodetection, and within each, a single square *A*rea *O*f *I*nterest (AOI) spanning 9.0 mm^2^ was identified. Each AOI was further parcelated into fields of interest each equal to 37638.6 μm^2^ and representing the entire area of a 40× photomicrograph. Three consecutive photomicrographs, one of each fluorescence channel starting with red (TRITC for AT1), followed by green (FITC for TH), and ending with blue (DAPI for nuclei) were captured for each section at predetermined and fixed exposure settings for each fluorophore (TRITC at 500 ms; FITC at 2 s; DAPI at 200 ms) using a Nikon Eclipse E600 microscope equipped with a Cool*SNAP*™ EZ monochrome CCD camera (Photometrics). For each patient, all neurons immunopositive for TH were counted in one AOI within matrix and one AOI within nigrosome 1 of one section, i.e., within the same AOIs that were used for AT1 detection and intensity measurement. For a neuron to be counted it had to be TH+ and contain a DAPI-labelled nucleus. The TH+ neuron counts (density) are reported as TH+ neurons/mm^2^.

In order to measure the intensity of AT1 expression in dopamine neurons, all TH- immunopositive neurons (FITC fluorescence) within the nigrosome 1 AOI and within the matrix AOI in each photomicrograph had their perimeter manually outlined with an area-polygon selection tool in Nikon software NIS-Elements BR3 software (Nikon). To quantify the intensity of AT1 in each whole dopamine neuron profile, the outline of each dopamine neuron profile (FITC channel) was copied and pasted into the TRITC fluorescence channel exactly in the same location on the photomicrograph as that for the FITC channel. Similarly, the intensity of nuclear AT1 was measured by outlining the nuclei (DAPI fluorescence) of dopamine neurons and copying and pasting the resulting outline into the TRITC channel. For the AT1-related fluorescence intensity quantification used as an index of the AT1 abundance, the sum intensity in each neuron within the AOI and within each nucleus were averaged for the group of neurons in the matrix and in nigrosome 1 for comparisons of AT1 intensity per neuron and per nucleus in these two nigral regions. The number of dopamine neurons and their nuclei examined within AOIs was within the following ranges: *i)* in AMC cases from 41 to 154 in matrix and from 39 to 140 in nigrosome 1; *ii)* in prePD cases from 71 to 147 in matrix and from 21 to 47 in nigrosome 1; and *iii)* in PD cases from 10 to 151 in matrix and from 10 to 44 in nigrosome 1. In total, 2086 dopamine neurons were analyzed using immunofluorescence intensity measures in order to estimate AT1 expression levels in dopamine neurons and their nuclei.

### Semiquantitative assessment of the degree of neuronal stress in nigral TH- and AT1-immunoreactive dopamine neurons and AT1 detection at the nuclear membrane

The numbers of nuclei in pigmented neurons that contained both Nox4/AT1 and StressMarq/AT1 immunoreactivity, or contained neither were counted in three fields captured at 40× magnification from both matrix and nigrosome 1 in each patient. The numbers of dopamine neuron nuclei examined within these three fields were as follows: *i)* in AMC cases 104; *ii)* in prePD cases 81; and *iii)* in PD cases 179. In total, 147 dopamine neurons were analyzed to detect the presence of nuclear Nox4 and 103 dopamine neurons were analyzed to detect the presence of nuclear StressMarq. To determine correlations between the presence of Nox4 and StressMarq in AMC, versus prePD, versus PD, the percentage of Nox4 and StressMarq immunoreactive nuclei was determined and expressed as a percentage of affected neurons. To determine if the nuclear AT1 was associated with nuclear membrane, selected tissue sections were co-immunoreacted with antibodies targeting AT1 and nucleoporin 62 (Nup62) and to assess the degree of injury associated changes in dopamine neurons, one tissue section of substantia nigra from each patient was immunoreacted with active caspase-3 antibody allowing detection of the intensity and sub-cellular localization of caspase-3 in matrix and nigrosome 1 relative to neurological and neuropathological change.

### Statistical analysis

Data are expressed as mean ± SEM with significance set at p ≤ 0.05. To assess differences among groups, data were analyzed by one way ANOVA followed by Student-Newman-Keuls post hoc analysis in SigmaPlot 11 scientific graphing and data analysis program (Systat Software Inc.).

## Results

### AT1 in dopamine neurons, microglia, astrocytes, and vascular smooth muscle cells in the substantia nigra of neurologically and neuropathologically normal patients (AMC) as well as in prePD and PD patients

**Figure 2:**
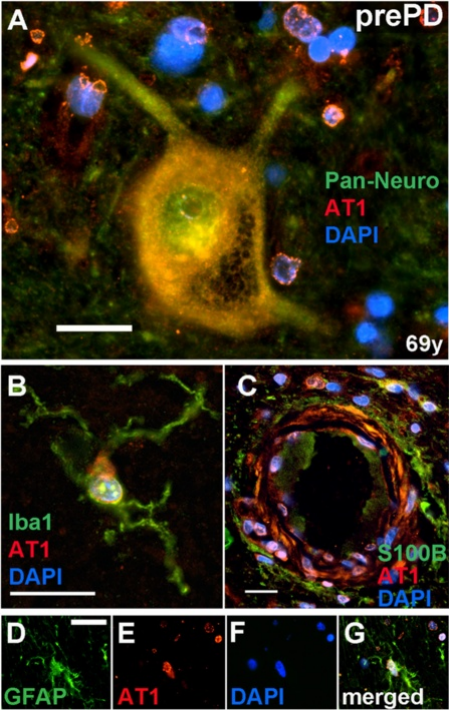
**Intracellular AT1 in neural and vascular cells of the adult human substantia nigra.** AT1 is shown to be highly expressed in nigral dopamine neurons **(A)**, in Iba1-positive microglia **(B)**, and in S100B- **(C)** and GFAP-immunoreactive astrocytes **(D-G)** of a 69 years old prePD patient. AT1 is also detected in vascular smooth muscle cells in the tunica media (*i.e.,* the medial layer) of the blood vessel wall **(C)**. Scale bars are 20 μm.

In all patients examined, irrespective of their health status (AMC, prePD, or PD), we found AT1 in all dopamine neurons. Adding prominence to the brain’s AT1 expression profile is the fact that AT1 was also expressed in all neurons as evidenced by the AT1 immunoreactivity present in neurons that were immunopositive for a Pan Neuronal marker, but were not pigmented and/or immunopositive for TH. An example of such neuronal AT1 expression is illustrated in a nigral section from a 69-year old prePD patient (Figure 2A). In addition, in all patients examined, AT1 immunoreactivity was present in both Iba1-immunoreactive microglia (Figure 2B) and S100B- and GFAP-immunoreactive astrocytes (Figure 2C-G). AT1 expression was also noted in vascular smooth muscle cells of the blood vessels within substantia nigra (Figure 2C). The AT1 expression pattern identified with the primary anti-AT1 antibody from Santa Cruz was identical to that detected in select sections with an alternative anti-AT1 antibody purchased from Sigma (data not shown).

### Loss of dopamine neurons in nigrosome 1 and in the matrix correlates with progression from AMC to prePD to PD

**Figure 3:**
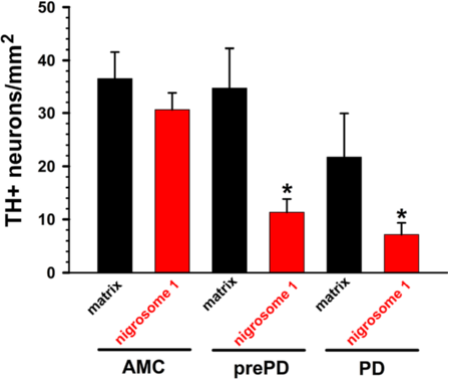
**The number of TH-positive neurons counted in matrix and nigrosome 1 areas of SN in neurologically intact individuals (AMC) and in prePD and in PD patients.** TH+ neurons were examined in two 9.0 mm^2^ areas of the SN, *viz.,* in the matrix region, which is calbindin-rich, and nigrosome 1, a calbindin-poor region. Data are expressed as mean ± SEM; _*_ denotes significant differences (p < 0.05) between nigrosome 1 in prePD and in PD as compared to AMC. The number of TH+ neurons in the matrix was not significantly altered with clinical diagnosis.

The numbers of dopamine neurons in nigrosome 1 of prePD and in PD patients were markedly less (p = 0.002 and p < 0.001, respectively) than corresponding dopamine neuron counts in nigrosome 1 in AMC patients. Unlike the findings in nigrosome 1, the numbers of dopamine neurons in the matrix did not correlate with clinical diagnosis, although there was a trend toward reduction in these neurons in PD patients (Figure 3). These differences in cell loss between the matrix and nigrosome 1 suggest that the AT1 levels, distribution, and actions might also be differentially affected in neurons in these two nigral compartments, which is consistent with the view that the earliest signs of nigral dopamine neuron loss in Parkinson’s occur in the nigrosome 1 [23].

### Total cellular AT1 expression in nigral dopamine neurons is reduced with disease progression, but nuclear AT1 in such cells in nigrosome 1 is unchanged

**Figure 4:**
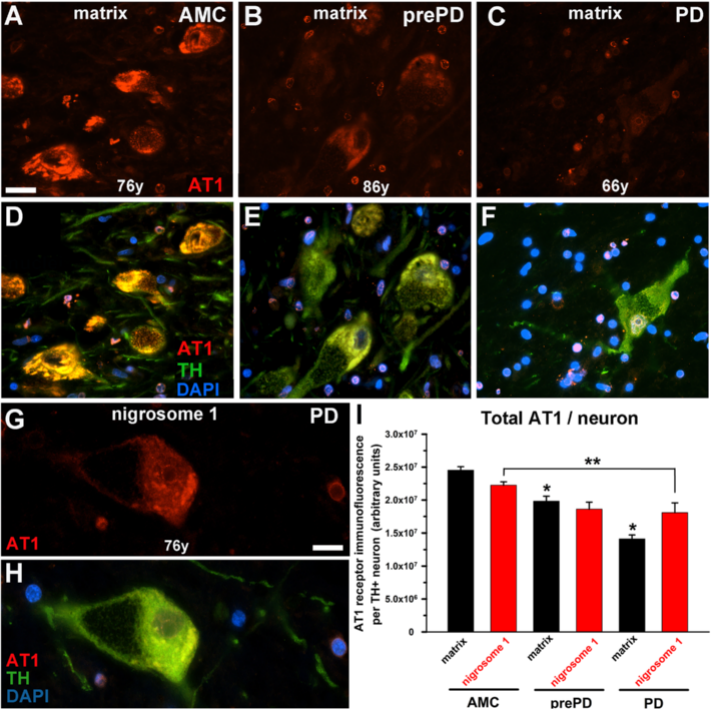
**Total cellular AT1 in SN dopamine neurons. (A-F)** Immunodetected AT1 in the SN matrix (calbindin-rich area) from neurologically intact patients (AMC), from prePD patients, and from PD patients. **(G** and **H)** show an example of AT1 expression in nigrosome 1 (calbindin-poor area). Scale bar in **(A-F)** is 20 μm and in **(G** and **H)** is 10 μm. **(I)** Quantification of AT1 immunofluorescence in TH-immunopositive dopamine neurons in the SN matrix and in nigrosome 1 as well as images **(A-F)** clearly illustrate dramatic reduction in the AT1 expression in nigral dopamine neurons (most profound in the matrix) as disease progresses. In panel **(I)**, a single asterisk identifies this AT1 immunofluorescence intensity measure as significantly different from the intensity measures in the other two matrix groups. The double asterisk indicates a significant difference between nigrosome 1 of AMC and PD.

The observation by Ge and Barnes [21] of a significant reduction in ligand binding to the AT1 in tissue samples from the putamen, caudate, and substantia nigra of Parkinson’s patients as compared to such ligand binding in tissues from neurologically normal control patients aroused our suspicion that neurons might substantially contribute to such changes in the AT1 expression. We found that the intensity of AT1 immunoreactivity in surviving dopamine neurons in both matrix (Figure 4A-F) and in nigrosome 1 (Figure 4G and H) declined stepwise from neurologically intact patients in a disease-related fashion (AMC>prePD>PD). In fact, in dopamine neurons in nigral matrix of some patients with advanced PD, AT1 expression was almost non-existent (Figure 4C and F). A comparison of the expression of AT1 in dopamine neurons in nigral matrix and nigrosome 1 of tissue from control patients, or from either prePD or PD, demonstrated that the total AT1 immunofluorescence intensity per dopamine neuron was substantially reduced (p < 0.001) (Figure 4I).

**Figure 5:**
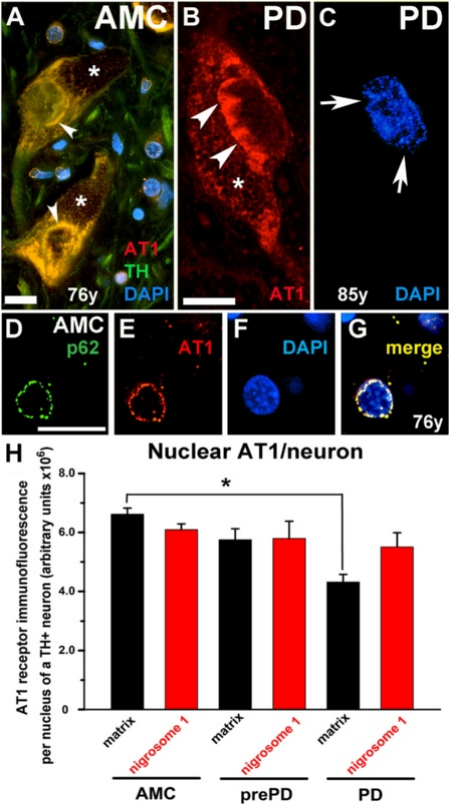
**AT1 accumulates at the nuclear envelope and within nuclei.** In dopamine neurons, AT1 is typically localized to the cytoplasm, to the perinuclear structures (arrowheads), and to the nuclei **(A)**. In addition, buildup of the AT1 within nuclei identified by arrowheads in **(B)** often coincides with irregularly-shaped nuclei with chromatin irregularities identified by the arrows **(C)**. In neurons, as well as in some non-neuronal cells as depicted here, AT1 co-localizes with nucleoporin 62 (p62), a component of the nuclear pore central channel **(D-G)**. Quantification of AT1 immunofluorescence within nuclei of TH+ neurons in neurologically intact individuals/AMC and in prePD and in PD patients **(H)** shows selective changes in AT1 corresponding to the disease progression. Notably, the abundance of nuclear AT1 in nigrosome 1 remains constant as disease advances whereas, in the matrix, the abundance of nuclear AT1 is gradually reduced as a function of disease progression following the trajectory of the total AT1 decline within the entire neuron (Figure 4I). Scale bars are 10 μm. Asterisks in (**A** and **B**) identify neuromelanin and the asterisk in **(H)** identifies a significant difference between matrix of AMC and PD.

**Figure 6:**
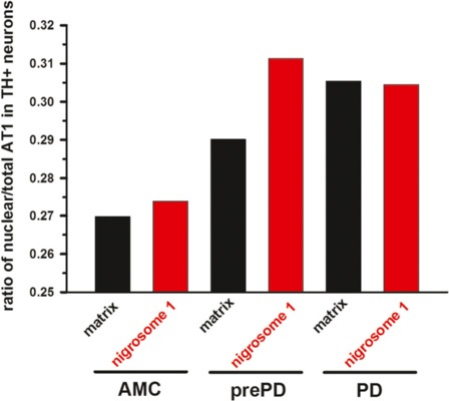
**Ratio of nuclear to total AT1 in TH+ neurons.** The ratio was calculated by dividing the average AT1 immunofluorescence intensity measured in the nuclei of TH+ neurons by that measured from the entire TH+ neurons in neurologically intact individuals/AMC and in prePD and in PD patients.

Although AT1 immunoreactivity was most evident in dopamine neurons, it was also present in non-TH-immunoreactive neural cells, and this immunoreactivity followed a most-to-least order from perinuclear, to cytoplasmic, to intranuclear (Figure 5A-B). Many nuclei of dopamine neurons, particularly in tissues from PD and prePD patients, exhibited AT1 accumulations, which appeared to penetrate the nucleus and coincide with irregularly-shaped nuclei (Figure 5C). In addition to this apparent intranuclear localization, AT1 was also noted within the nuclear envelope as evidenced by co-localization of AT1 immunoreactivity with an integral subunit of the nuclear pore complex, nucleoporin p62 [29,30] (Figure 5D-G). AT1 co-localized with p62 in neurons and numerous smaller cells in all AMC, prePD, and PD cases examined. Quantification of AT1 immunofluorescence within nuclei of dopamine neurons in neurologically intact individuals/AMC and in prePD and in PD patients (Figure 5H) showed that the density of nuclear AT1 in nigrosome 1 remains constant as disease progresses whereas, in the matrix, the abundance of nuclear AT1 is gradually reduced as a function of disease progression following the trajectory of the total AT1 decline within the entire neuron (Figure 4I). Consequently, the ratio of nuclear AT1 to total AT1 (nuclear + cytoplasmic + membrane) in dopamine neurons of nigrosome 1 (Figure 6) increased stepwise from AMC (0.274) → prePD (0.311) → PD (0.305). The increase in the ratio was more gradual in the matrix (Figure 6), as compared with the nigrosome, demonstrating the following pattern of increases: AMC (0.270) → prePD (0.290) → PD (0.305).

### Intranuclear expression of Nox4 in dopamine neurons is associated with intranuclear oxidative damage to nucleic acids and activation of caspase-3

**Figure 7:**
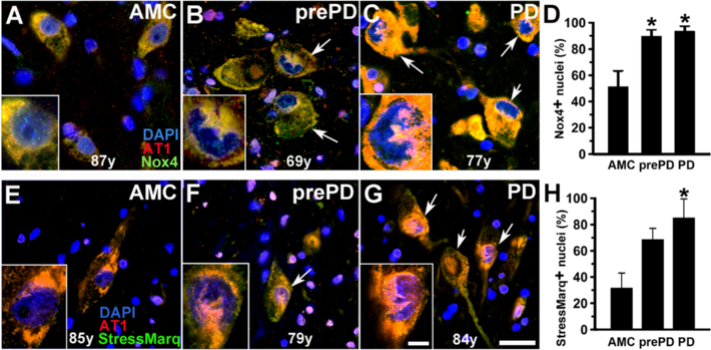
**Intranuclear expression of Nox4 is associated with intranuclear oxidative damage to nucleic acids.** Nox4 expression favors nuclear localization in dopamine (pigmented) neurons in prePD and PD patients as compared to AMC individuals **(A-C)**. Quantification of the percent of dopamine neuron nuclei that are Nox4+ **(D)**. Oxidative stress-induced damage to the nucleic acids is demonstrated by a shift of immunohistofluorescently-detected StressMarq signature of oxidized guanosine (8-OH guanosine) into the nuclei of dopamine neurons **(E-G)** – quantified in panel **H**. Scale bar is 20 μm. Arrows point to neurons with intranuclear expression of Nox4 or presence of StressMarq-identified 8-OH guanosine. Asterisks in (**D** and **H**) identify significant differences from AMC.

Because of our evidence demonstrating a direct link between AngII stimulation of the AT1 and activation of Nox4-dependent generation of superoxide in neural stem cells [12], together with evidence by others of prominent nuclear localization for Nox4 and Nox4-mediated superoxide generation [31,32], we explored the possibility that analogous interactions between the AT1 and Nox4 might occur within the nuclei of dopamine neurons in substantia nigra of Parkinson patients. Moreover, we found co-existence of AT1 and Nox4 immunoreactivity in DAPI-labelled nuclei of dopamine neurons in nigral tissue from AMC, prePD, and PD patients (Figure 7A-C). Quantification of the percent of such dopamine neuron nuclei that were Nox4 immunoreactive showed that 51% of the AMC nuclei were Nox4 positive, whereas in prePD and in PD the nuclear Nox4 was significantly increased and noted in 89% and in 93% of the nuclei examined, respectively (Figure 7D). Such increases in the nuclear Nox4 expression were accompanied by similar increases in oxidative stress-induced damage to neuronal nucleic acids as demonstrated by elevated immunodetection of StressMarq signature of oxidized guanosine (8-hydroxy guanosine; 8-OH guanosine) in the nuclei of dopamine neurons (Figure 7E-G) with a progressive pattern: 31% for AMC < 68% for prePD < 82% for PD (Figure 7H). Taken together our measures support the idea of nuclei-delimited pathologic changes involving AT1 → Nox4 → superoxide-induced nucleic acid damage, which coincide closely with the pathologic progression in the substantia nigra from AMC → prePD → PD.

**Figure 8:**
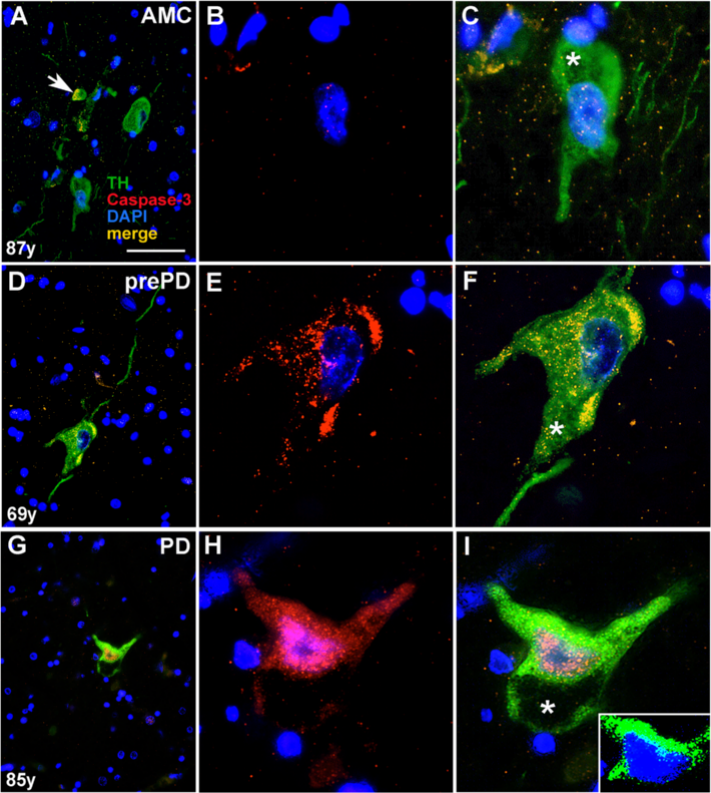
**Activation of caspase-3 corresponds to severity of PD.** Immunodetection of active caspase-3 in TH-immunoreactive dopamine neurons in the SN of AMC **(A-C)**, prePD **(D-F)**, and PD **(G-I)** patients. In an AMC case **(A)**, arrow identifies a dopamine neuron immunopositive for active caspase-3 in an area otherwise containing little active caspase-3. In addition, depicted are magnified views of subcellular localization of active caspase-3 in reference to the nuclei **(B, E, H)** as well as of individual neurons in merged images **(C, F, I)**. Panel **(I)**, contains an additional inset at the magnification of the main panel **(I)** illustrating vividly the boundaries of the DAPI-positive nucleus within a TH-immunoreactive dopamine neuron to more clearly depict the extent of active caspase-3 labelling within the nucleus. Scale bar equals 50 μm (**A**, **D**, and **G**) or 18 μm (**B**, **C**, **E**, **F**, **H**, **I**, and inset in **I**). Asterisks in (**C**, F, and **I**) identify neuromelanin.

The degree of activation of the AT1 → Nox4 → superoxide cascade correlated with immunodetection of heightened levels of activated caspase-3, a surrogate for an irreversible step in the apoptotic cell death pathway. In the aged human brain, we have observed a continuum of active caspase-3 phenotypes, such that most of the neurologically normal control individuals demonstrated some, albeit typically low, levels of active caspase-3 in dopamine neurons (Figure 8A-C), while in prePD and PD cases dopamine neurons displayed more intense fluorescence of active caspase-3 (Figure 8D-I). The distribution of active caspase-3 between the cytoplasmic and the nuclear compartments was also heterogeneous (Figure 8) indicating that age-related stresses affect caspase-3 activation status in humans.

## Discussion

Here, we report that dopamine neurons from intact substantia nigra of neurologically and neuropathologically normal patients (AMC) differ substantially with respect to the levels of AT1 expression as well as intranuclear expression of superoxide-generating Nox4, nuclear oxidative stress, and cellular activation of caspase-3 from clinically asymptomatic patients, who had only modest dopamine cell loss, but had Braak stages that were consistent with a neuropathological diagnosis of PD (referred to here as prePD), and from advanced PD patients. We found that the total cellular AT1 levels were reduced in dopamine neurons of prePD patients as compared to age-matched neurologically normal controls (AMC). Previously, Ge and Barnes [21] demonstrated reduced radiolabelled AngII binding to the AT1 in tissue extracts from caudate nucleus, putamen, and substantia nigra of Parkinson patients with advanced disease compared to neurologically normal control patients. We established that this general loss of AT1 in substantia nigra is largely due to the reduction of AT1 in dopamine neurons, which led us to speculate that neurons in other basal ganglia structures, namely the caudate and putamen, might also experience a reduction in AT1. In our study, tissue from clinically- and neuropathologically-confirmed PD patients had greater cell loss and greater decline in the total cellular levels of AT1 in surviving dopamine neurons in both the calbindin-rich matrix and in the calbindin-poor nigrosome 1 than was observed in dopamine neurons from prePD patients. This pathology-associated decline in AT1 and the accompanying increase in Nox4 from AMC to prePD and finally to PD is suggestive of a progressive loss of dopamine neurons in nigral matrix and nigrosome 1 due to excess Nox4 generation in the nucleus of dopamine neurons and a consequent increase in production of superoxide at levels that precipitate nucleic acid damage leading to neuronal dysfunction and death.

Even though a combination of insufficient tissue sample to perform unbiased stereological counts for TH+ neurons necessitated counts of all nigral TH+ neurons from just one section per patient and the number of available patients (particularly prePD, n = 3) resulted in relatively high standard errors, the counts of TH+ neurons obtained corresponded closely with the original nigrosome/matrix principles defined by Damier and colleagues [23], in which loss of nigrosome 1 dopamine neurons is faster than the loss of such neurons in the matrix. In that vein, we observed that in PD as well as in prePD the density of TH+ neurons was markedly and significantly lower in nigrosome 1 as compared with matrix. In addition, and in agreement with the Damier principle [23], in our patients the density of TH+ neurons was the same in the matrix and nigrosome 1 in AMC and followed a trend of gradual decline with the disease progression. Our observation that the decline in the AT1 expression in the matrix, a structure with a relative to nigrosome 1 sparing of dopamine neurons in PD (Figure 4 and [23]), is greater than that in the more affected nigrosome 1 where we detected a significant loss of TH+ neurons in both prePD as well as in PD cases, suggests that probability that neurons survive increases in association with lower total (Figure 4) as well as nuclear (Figure 5) AT1 expression.

**Figure 9:**
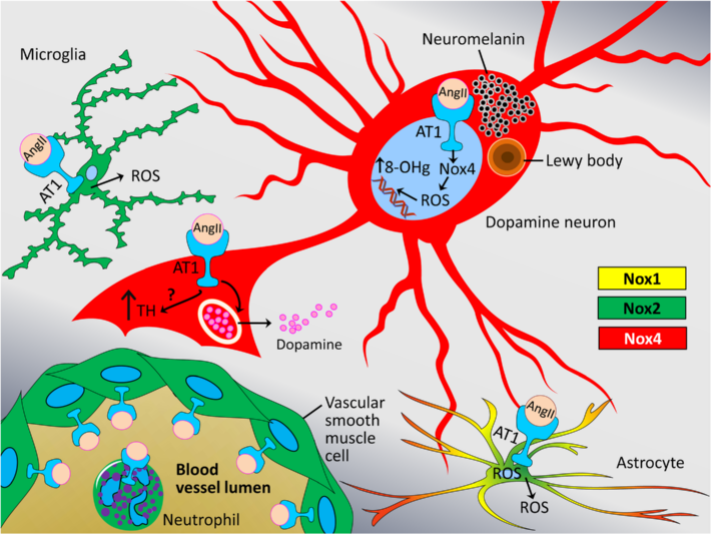
**A cell type-dependent model of interactions between AT1 and Nox1, 2, and 4 in the human substantia nigra.** The AT1 is broadly expressed in the CNS in astrocytes, microglia, and is particularly abundant in neurons, including dopamine neurons where, as we demonstrate here, the AT1 is found not only on the plasma membrane, but also intracellularly associated with the membranes of the endoplasmic reticulum, other cytoplasmic structures, nuclear membrane, and AT1 is found inside the nuclei themselves. The AT1 is also expressed by the vascular smooth muscle cells and by neutrophils and macrophages, raising the possibility that brain RAS activities can be influenced by the entry of peripheral monocyte-derived cells that are rich in AT1 and Nox2. The nuclear AT1, which we demonstrate here to be co-localized with nuclear Nox4, an event which frequency increases with disease progression, induces intra-nuclear production of reactive oxygen species (ROS, superoxide and hydrogen peroxide) leading to an increase in nucleic acid oxidation determined by increased levels of oxidized 8-OH guanosine (8-OHg). This complex landscape of AT1-Nox interactions, when balanced serves to maintain tissue homeostasis and normal levels of dopamine, but in chronic disease such as Parkinson’s the AngII/AT1/Nox4 axis might become overactive and lead to deleterious nucleic acid lesions that destabilize DNA and impair the transcriptional machinery in the affected neurons. Taken together, our findings suggest need for additional studies of these interactions toward designing therapies that restore healthy balance between the injurious and physiological functions of AT1 and Nox4 and by doing so moderate progression or prevent onset of neurodegenerative diseases. Legend: neurons – Nox4 (red), microglia – Nox2 (green), and astrocytes – mixture of Nox1 (yellow), Nox2 (green), and Nox4 (red).

While, overall, the total levels of AT1 decline in dopamine neurons from AMC to prePD to PD, dopamine neurons in nigrosome 1 differ in that the ratio of nuclear AT1 to total AT1 (nuclear + cytoplasmic + membrane) increased stepwise from AMC to prePD to PD. The mechanistic underpinnings of the proportional increase in nuclear AT1 in surviving dopamine neurons in nigrosome 1 of prePD and PD patients might be related to accompanying elevation in nuclear expression of superoxide-generating Nox4, which in turn may explain our finding of increased oxidative damage to DNA as measured by elevated 8-OH guanosine (summarized in Figure 9) and ongoing caspase-3-mediated proteolysis and cell death. The increase in the percent of Nox4+ and StressMarq + nuclei in the context of unchanging (or even declining in the matrix) AT1 expression might represent a compensatory mechanism, by which cellular stresses imposed on the dopamine neurons that might be directly or indirectly related to changes in AT1 expression promote increases in Nox4, which is known to be transcriptionally activated by stress and AngII [12].

Nuclear localization of Nox4 has been reported from other cell types such as human vascular endothelial cells [33] and from cardiomyocytes, in which Nox4 causes histone deacetylase 4 (HDAC4) cysteine oxidation, leading to HDAC4 exit from the nucleus and cardiac hypertrophy [31]. Within nuclei, Nox4 has been reported to localize to speckle domains, i.e., nuclear domains in the interchromatin regions of the nucleus, adjacent to highly transcriptionally active regions, suggesting that Nox4-generated superoxide might be regulating proteins involved in pre-mRNA splicing in these unique nuclear compartments [32], underscoring the potential for Nox4 to regulate the dynamics of the nuclear gene expression machinery. In addition to its nuclear distribution, Nox4 is often found in the mitochondria in various cell types including neurons [34]. In cardiomyocytes, hypertrophic stimulation increases production of Nox4, which is constitutively active as its activity is principally controlled by its expression levels, which leads to increased superoxide production and mitochondrial dysfunction – events that culminate in apoptosis of cardiomyocytes [35]. An example of the deleterious role of Nox4 in the brain comes from studies of ischemic stroke, where Nox4 is a major source of oxidative stress, a finding supported by the observations that pharmacological as well as genetic inhibition of Nox4 in a mouse model of stroke prevents oxidative stress and neurodegeneration [36]. In addition to its role in oxidation, Nox4 promotes inflammation as reported by Ginnan and colleagues in smooth muscle cells where Nox4 is required for interleukin 1β-mediated activation of protein kinase C and of c-jun N-terminal kinase [37].

While Nox4 can drive pathogenic pathways when overactive, physiological activation of Nox4 is important for maintenance of many cellular functions, including transcription and proliferation. In that regard, the changes noted here in AngII/AT1/Nox4 and the implicit generation of superoxide, and in 8-OH guanosine and its signature nucleic acid oxidation may have been elicited in an attempt to protect these dopamine neurons, and/or to stimulate regeneration, as has been reported in the leukocyte-dependent regeneration of zebra fish tail fin [38]. Such regeneration is dependent on production of hydrogen peroxide, which in addition to its antiseptic actions, sets up a hydrogen peroxide gradient across the margin of the wound, signaling rapid recruitment of leukocytes, restoration of the epithelial barrier between the injured tissue and the outside environment and pathogens, and stem cell proliferation for fin genesis [38]. However, as exemplified in neurodegenerative diseases in general, such mechanisms may initially be elicited toward restoration of neuronal resilience [28,39] as we have shown previously for AngII/AT1/Nox4-mediated neural stem cell proliferation [12], but when neuronal stress is prolonged overtime these mechanisms may lead, as our findings indicate here, to oxidative damage and loss of dopamine neurons and the frank neurological and neuropathological manifestations of neurodegenerative diseases such as Alzheimer’s and Parkinson’s.

To investigate the potential for apoptotic neuronal cell death in our AMC, prePD, and PD patients, we determined caspase-3 activation in dopamine neurons across these disease stages. There are strong similarities between historical findings of a temporal relationship between a progressive rise in the levels of cleaved caspase-3 in both the cytoplasmic as well as the nuclear compartments in nigral dopamine neurons in rats injected intrastriatally with 6-OHDA [40,41] and our findings of a stepwise AMC<prePD<PD increase in cleaved caspase-3 in cytoplasm of nigral dopamine neurons that culminated in caspase-3 expression in both cytoplasm and nucleus in frank disease. These historical findings [40,41], together with our findings in human tissue are indicative of an AngII/AT1/Nox4-superoxide axis that contributes to, and thus may be responsible for, the progressive dysfunction and loss of dopaminergic neurons that underlie the neuropathological changes and neurological failures characteristic of Parkinson’s.

Inquest into brain angiotensin-regulated pathways raises a question of why components of RAS are expressed in neurons. Several lines of evidence point to counter-regulation between RAS and dopamine as a principal reason, where, as we hypothesize, the increase in AngII signaling via AT1 might directly promote dopaminergic neurotransmission by increasing the activity of TH, as was demonstrated outside the brain in organs such as the kidney [42]. Additional support for angiotensin↔ dopamine counterregulation comes from experiments in rats, where depletion of dopamine with an inhibitor of TH, reserpine, resulted in an increase in AT1 at both mRNA and protein levels [43]. In parallel experiments in which similar increases in AT1 were achieved with 6-OHDA administration, co-administration of L-dopa prevented such increases in AT1 [43]. In our study, in contrast to the prePD patients who were not treated with L-dopa, in L-dopa-medicated advanced PD patients, the L-dopa treatment might have affected the AT1 expression profiles. One could speculate that such effects could potentially include L-dopa promoting lowering of membrane and cytoplasmic AT1 expression while lacking an effect on the nuclear AT1 – a pattern of AT1 expression changes that we have demonstrated in the current study. Further studies of the L-dopa effects on the RAS are needed to illuminate these important angiotensin↔dopamine counterregulatory mechanisms in the human brain. Such studies should also consider potential contributions of other biological processes on the regulation of AT1 expression. In addition to L-dopa-regulation of AT1 levels, other molecular entities and biological processes affect AT1 levels. For example, elevation of AngII/AT1 also occurs in rodents with: *i)* advancing age [17], *ii)* reduction in estrogen modeling menopause [44], and *iii)* hypoperfusion of the brain [45]. Central activity of AngII has also been reported to be elevated in conditions that compromise blood–brain barrier (BBB), such as neurogenic hypertension, permitting circulating AngII access to the CNS [46]. Such effects of the circulating AngII on the CNS were also demonstrated in rats with mannitol-mediated experimental disruption of the BBB, which resulted in circulating AngII gaining access to the rostral ventrolateral medulla; there AngII increased activation of dopamine neurons assayed by an increase in c-Fos immunoreactivity in TH-immunopositive neurons [47].

The idea of altering the activity of RAS for therapeutic benefit in Parkinson’s was first tested in Melbourne by demonstrating in a prospective study that the angiotensin converting enzyme (ACE) inhibitor, perindopril, reduces levodopa-induced dyskinesias [48]. Subsequently, progress in elucidating the potential role of AngII/AT1 interactions in Parkinson neuropathogenesis has been greatly aided by the development of MPTP- and 6-OHDA-rodent models of Parkinson’s [4,18]. Treatment of rodents with blockers of AT1 (ARBs) before administration of either MPTP or 6-OHDA prevents neurotoxic effects of these agents through less microglial activation, less generation of NADPH oxidase (e.g., Nox4) and therefore less superoxide production, and less neuronal dysfunction and loss, suggesting that ARBs promote dopamine neuron resilience and survival. The insights gained from the rodent studies have now been validated in humans by Lee and colleagues who report in a study of 65,000 neurologically normal hypertensive patients that use of high cumulative doses of ARBs is associated with reduced risk of developing Parkinson’s when compared to the use of β blockers [49]. The beneficial effects of ARBs on human CNS neurons extend beyond Parkinson’s. As a matter of fact, among Alzheimer patients treated with ARBs the incidence of Alzheimer is less and there is less Alzheimer neuropathology [50,51]. Moreover, cognition is improved [50,52].These findings in patients, together with studies of animal models of Parkinson’s and Alzheimer’s [4,18,53], suggest that downregulation of the AngII/AT1-driven cascades may, in general, confer neuronal protection. Whether such interactions are part of the initiation and progression of the neuropathological changes in dopamine neurons that are associated with Parkinson’s is, at present, unclear. Furthermore, if such events are part of Parkinson’s pathogenesis, whether and/or how intervention to modify AngII/AT1 interactions may circumvent or delay onset of Parkinson neuropathogenesis is unknown. To our knowledge, this present report is first to demonstrate the cellular and subcellular distribution of AT1 and Nox4 relative to Parkinson’s disease progression.

Our examination of nuclear morphology showed specific changes that correlated with disease progression. The nucleus is the largest organelle in a dopamine neuron and has intricate connections to the cytoskeleton that maintain topographical and functional nucleo-cytoskeletal interdependence [54]. In addition, changes in nuclear shape and size are associated with diseases such as cancer as well as with aging [55,56]. We have noted a potential disruption of normal nuclear morphology in dopamine neurons coincident with accumulations of aberrantly-expressed assemblies containing nuclear deposits of AT1 and Nox4 infiltrating deep into the nuclei of affected dopamine neurons. In most such affected neurons, these AT1/Nox4 accumulations infiltrate nuclei from only one side along the nuclear long axis. Whether such nuclear accumulations disrupt structure-dependent molecular interactions and gene expression profiles by destabilizing normal regulation of nuclear processes such as molecular crowding, which controls diffusion and interactions of nuclear proteins with heterochromatin [57,58], and thereby accelerate the advance of neurodegenerative disease pathology and neuronal dysfunction, is unknown. By analogy to our findings of AT1/Nox4 intranuclear accumulations, filamentous tau deposits that span the nuclear long axis and are referred to as “tau nuclear rods” have been reported from neurons in the striatum and in the cortex of Huntington patients and in the hippocampal neurons in Alzheimer patients [59]. Although tau nuclear rods are hypothesized by some to be harmless signatures of altered microtubule dynamics, other investigators propose that tau nuclear rods could compromise the integrity of the nuclear membrane promoting nuclear fragmentation and cell death [59]. The previously unrecognized patterns of proportional increases in nuclear AT1 in dopamine neurons in nigrosome 1 of prePD and PD patients that we observed might underscore a surge in neurooxidative and neuroinflammatory processes favoring increased intranuclear stress and oxidative damage to nucleic acids leading to promulgation of untoward transcriptional changes. Hypothetically, such aberrant nuclei could arise from the disease process-related topographic modulation of the nuclear shape – a feature that might serve as a surrogate of pathologic progression in Parkinson’s. Future studies of AT1/Nox4 nuclear buildup should examine changes to the nuclear geometry and nucleo-cytoskeletal dynamics using quantitative nuclear morphometry and interrogate these nuclear features for association with changes in gene expression patterns.

Unlike many studies that report expression changes from only subjective ranking or from small numbers of cells, we have dedicated a substantial effort to assure that AT1 expression was measured in a highly controlled and reproducible fashion in a large number of TH+ neurons. The quantification method employed by us utilized Nikon Element’s data analysis software intensity measurements that have been used and confirmed against other methods for protein expression analysis (Western immunoblot and ELISA) by us and other groups and published in a number of reports [26,28,60]. In addition, detailed examination by two blinded evaluators of the computer-generated AT1 signal intensity measured corresponded reliably with the visual examination of the same cells in corresponding photomicrographs. Despite a series of important changes in expression and localization of AT1 that we found to be associated with disease progression, our study has limitations including low numbers of prePD patients (n = 3) and use of immunohistofluorescence as a sole method for AT1 quantification. Practical reasons including the fact that presymptomatic patients demonstrating Braak stages and dopamine neuron loss consistent with early PD are difficult to find and that the brain tissues from these patients were available only in the form of paraffin-embedded tissue blocks necessitated the present study design. Future studies will focus on identification of a larger cohort of prePD patients in whom RAS-related biomarkers of early disease can be examined further using in addition to immunohistochemical methods, multiplex gene expression profiling and biochemical protein analysis approaches.

The evolutionary advantage of increasing AngII/AT1 activity might reflect the brain’s attempt to counteract the deficits in dopaminergic neurotransmission that accompany progression of Parkinson’s disease, and concurrently, via the AngII/AT1/Nox4 pathway increase neurogenesis as a measure to replace neurons being lost. Our current study and earlier investigations in animal models as well as by means of clinical studies with ARBs demonstrated a fundamental role for the AT1 in Parkinson’s and Alzheimer’s, but as of yet have not explored other molecular players in the complex network of brain RAS. These players include at least four other angiotensins (AngIII, AngIV, Ang_1–7_, and Ang_3–7_) in addition to AngII; another ACE, namely ACE2; as well as four additional receptors, namely AT2, AT4, Mas, and pro-renin receptors, extending substantially the RAS-related receptor field beyond the AT1 (for review see [61]). Although examination of these additional components of RAS was certainly beyond the scope of the current study, investigations that will take place during the next decade will offer novel insights into the complex regulation of the brain RAS and will engineer means for modulating RAS activity for disease-modification or perhaps even prevention in Parkinson’s, Alzheimer’s, and beyond.

## Conclusions

Using semiquantitative immunohistofluorescence, we found that AT1 protein expression in dopamine neurons of the human substantia nigra is reduced with the disease progression from AMC → prePD → PD in the calbindin-rich matrix and in the calbindin-poor nigrosome 1, where we observed greater loss of TH-immunopositive dopamine neurons. In all cases examined, the AT1 was primarily associated with the intracellular structures within the cytoplasm as well as with the nuclear envelope and nuclear pore complexes, as determined by colocalization of the AT1 with nucleoporin p62, and deep within the nuclei of dopamine neurons. Although the nuclear AT1 in dopamine neurons located in the matrix demonstrated a similar loss of AT1 corresponding with the clinicopathological progression as was observed for the entire neuron AT1, the nuclear AT1 in the nigrosome 1 dopamine neurons did not show reduction and remained at levels characteristic of the neurologically and neuropathologically normal control patients. Therefore, we propose that such a nonphysiological shift in the cytoplasmic and nuclear AT1 distribution in favor of nuclear AT1 increases oxidative damage to DNA. Such a scenario is supported by the observed colocalization of the nuclear AT1 with superoxide-generating Nox4 and with 8-OH guanosine-detected oxidative damage to the nucleic acids. Disease progression from AMC → prePD → PD was also associated with increases in Nox4, oxidative damage to DNA, and activation of caspase-3 in the nuclei of dopamine neurons, giving support to the idea that an AngII/AT1/Nox4-superoxide axis contributes to the neurooxidative injury in dopamine neurons and underscoring the need for further studies of modifiers of the brain RAS activity for preventive and/or symptomatic treatment of neurodegenerative diseases.
